# Adult head circumference and the risk of cancer: a retrospective cohort study

**DOI:** 10.1007/s10552-025-01966-9

**Published:** 2025-02-06

**Authors:** Suhas Krishnamoorthy, Jonathan K. L. Mak, Kathryn C. B. Tan, Gloria H. Y. Li, Ching-Lung Cheung

**Affiliations:** 1 Department of Pharmacology and Pharmacy, The University of Hong Kong, 21 Sassoon Road, Pokfulam, Hong Kong, SAR China; 2 Department of Medical Epidemiology and Biostatistics, Karolinska Institutet, Stockholm, Sweden; 3 Department of Medicine, School of Clinical Medicine, The University of Hong Kong, Hong Kong, SAR China; 4 Department of Health Technology and Informatics, The Hong Kong Polytechnic University, Hong Kong, SAR China; 5 Laboratory of Data Discovery for Health (D24H), Hong Kong Science Park, Pak Shek Kok, Hong Kong, SAR China; 6 Hinda and Arthur Marcus Institute for Aging Research, Hebrew SeniorLife, Boston, USA

**Keywords:** Retrospective cohort study, Cancer, Head circumference, Epidemiology

## Abstract

**Purpose:**

Cancer-related genes and pathways have recently been implicated in a genome-wide meta-analysis of head size. In the current study, we aimed to evaluate the association between adult head circumference and the risk of cancer.

**Methods:**

This is a cohort study using data from the Hong Kong Osteoporosis Study, where 1,301 participants aged 27–96 years with head circumference measured between 2015 and 2019, and without a history of cancer, were followed up to 15 January 2024. Incident cancers were identified using electronic medical records from a territory-wide database. Hazard ratios (HR) and 95% confidence intervals (CI) were estimated using Cox proportional hazards regression, adjusting for age, sex, height, weight, education, smoking, alcohol drinking, physical activity, and family history of cancer, as well as accounting for familial clustering.

**Results:**

The median head circumference was 53 cm (interquartile range [IQR]: 51–54) and 54 cm (IQR: 53–55) for women and men, respectively. During a median follow-up of 6.9 years, 66 individuals were diagnosed with cancer. In the adjusted model, a larger head circumference was associated with an increased risk of any cancer (HR per cm increase: 1.17; 95% CI 1.00–1.36). Results remained similar when adjusting for waist-to-hip ratio instead of weight or when additionally adjusting for serum calcium and phosphorus levels. When stratified by cancer sites, head circumference was most strongly associated with colorectal cancer (HR per cm increase: 1.81; 95% CI 1.14–2.90) and prostate cancer (HR per cm increase: 1.58; 95% CI 1.16–2.16).

**Conclusion:**

Head circumference is positively associated with the risk of cancer independently of height, weight, and other cancer risk factors.

**Supplementary Information:**

The online version contains supplementary material available at 10.1007/s10552-025-01966-9.

## Introduction

Cancer is a leading cause of mortality globally, accounting for nearly 10 million deaths in 2022. [1]. As both the incidence and mortality of cancer continue to rise globally, the burden on populations and health systems is expected to increase [1]. While some cancers are strongly associated with risk factors, such as lung cancer and tobacco use, many malignancies remain poorly understood despite the conduct of numerous scientific studies. Improved understanding of risk factors is clinically important and may help reduce the cancer burden by enabling early prevention strategies [2].

Findings generated from human genetic association studies could have profound clinical implications [3]. Recently, the largest genome-wide meta-analysis of head size to date found that the genetic variants underlying human head circumference overlapped with cancer genes and biological pathways. In particular, gene set enrichment analysis of the head circumference variants found several enriched gene sets in various cancers and the p53, Wnt, and ErbB signaling pathways (Supplementary Table S1) [4]. In addition, a few studies have previously reported an association between head size at birth and the risk of developing certain types of cancer later in life [5–7]. However, whether adult head size could be a predictor of cancer risk has never been examined.

Given the connection between the genetic determinants of head size and cancer identified in previous research [4], we hypothesized that adult head circumference was associated with an increased cancer risk. Therefore, this study aimed to evaluate the association between adult head circumference and the risk of cancer overall and for specific cancer types in a cohort comprised of individuals from the Hong Kong Chinese population.

## Materials and methods

### Study population

The Hong Kong Osteoporosis Study (HKOS) is a prospective cohort established in 1995. The details of the cohort have been described previously [8]. Briefly, participants were randomly recruited from public roadshows and health fairs held in Hong Kong from 1995 to 2010. Family members were also recruited into the study for some participants with a low bone mineral density (BMD) at the hip or spine. 7,990 random participants were recruited along with 1,459 participants from 306 families at the baseline. Since 2015, the participants have been invited to attend in-person follow-up visits. Subjects were required to complete questionnaires (e.g., tobacco use, alcohol consumption) and undergo physical examinations (e.g., height, weight) at baseline. At in-person follow-ups, additional information was collected for 1,386 participants, including anthropometric data such as the circumference of the head, neck, waist, and hip. Among these participants, 356 individuals belonged to 106 distinct families. Follow-up of participants was done via linkage to the territory-wide electronic medical record, Clinical Data Analysis and Reporting System (CDARS), which is managed by the Hong Kong Hospital Authority (HKHA). The purposes of CDARS are for clinical management, research, and audit, and the data have been used for high-quality epidemiological studies [9–11].

In this analysis, the baseline date was defined as the date of in-person follow-up when head circumference was measured (between June 2015, and August 2019). We excluded participants who had been diagnosed with cancer before the baseline date (*n* = 60), those with missing head circumference and covariates (*n* = 13) as well as those with extreme head circumference, defined as above the 75th or below the 25th percentile by a factor of 1.5 times the interquartile range (*n* = 12). After exclusion, 1,301 participants were included in the final association analysis (Supplementary Fig. 1). All participants were followed until diagnosis of cancer, death, or 15 January 2024 (study end date), whichever came first.

### Head circumference and other covariates

During the in-person follow-up visits in 2015–2019, head circumference was measured by a trained research nurse or research assistant using a measuring tape and passing it around the head above the eyebrows and over the most posterior protuberance of the occiput.

We considered age, sex, height, weight, education, smoking status, alcohol drinking status, physical activity level, family history of cancer, and family cluster as the main covariates to be accounted for in the models. Height and weight were measured during in-person visits, and the socio-demographic and lifestyle variables were self-reported by questionnaires. Details of measurements of serum biomarkers of mineral metabolism in HKOS have been described previously [12].

### Cancer ascertainment

Data of incident cancer was retrieved from the CDARS using the International Classification of Diseases, Ninth Revision, Clinical Modification (ICD-9-CM) codes 140–208. According to the HKHA statistical report, CDARS captures > 80% of cancers in Hong Kong [13]. In our recent territory-wide lung cancer epidemiological study, the CDARS data was shown to closely align with the data reported by the Hong Kong Cancer Registry [9]. In addition to all cancers, we also examined the most common site-specific cancers in the sample, including breast cancer in women (ICD-9-CM code 174), lung cancer (including trachea; ICD-9-CM code 162), colorectal cancer (ICD-9-CM codes 153–154), stomach cancer (ICD-9-CM code 151), and prostate cancer in men (ICD-9-CM code 185).

### Statistical analysis

We compared baseline characteristics by sex-specific tertiles of head circumference using analysis of variance for continuous variables and chi-squared tests for categorical variables. The correlation between head circumference and other anthropometric measurements was evaluated using Spearman correlation. Hazard ratios (HR) and 95% confidence intervals (CI) for the incidence of any cancer and site-specific cancers were calculated using Cox proportional hazard regression. All models were evaluated for the proportional hazard assumptions using Schoenfeld residuals and no violations were found. Head circumference was analyzed as a continuous variable (per 1 cm and standard deviation [SD] increase) and a categorical variable using sex-specific tertiles. The models were first adjusted for age and sex (model 1) and further adjusted for weight, height, education, smoking, drinking, physical activity, and family history of cancer (model 2). As some individuals within the cohort were related, we accounted for familial clustering in model 3 using cluster-robust standard errors. A penalized regression spline was used to visualize the dose–response relationship between head circumference and the risk of any cancer. We also performed stratified analysis by baseline age (< 65 vs. ≥ 65 years), sex (women vs. men), BMI (< 25 vs. ≥ 25 kg/m^2^), and drinking status (never-drinker vs. ever-drinker) to explore potential effect modification. As a sensitivity analysis, we adjusted the models by waist-to-hip ratio instead of weight to reduce potential confounding by adiposity (model 4). Furthermore, since head circumference is largely determined by cranial bone size and could be correlated with calcium and phosphorus [14], we further included serum calcium and phosphorus levels as covariates in the models (model 5; *n* = 1,158). All statistical analyses were performed using R version 4.3.2.

## Results

The descriptive statistics of the 1,301 study participants are shown in Table 1. The mean age was 57.8 years and 1032 (79.3%) were women. Head circumference was approximately normally distributed (Supplementary Fig. 2), with a median of 53 cm (interquartile range [IQR]: 51–54 cm) in women and 54 cm (IQR: 53–55 cm) in men. Individuals with a larger head circumference were more likely to be younger (*p* = 0.049), taller (*p* = 0.005), have a higher weight (*p* < 0.001), have a higher education (*p* < 0.001), and have higher serum calcium (*p* = 0.002) and phosphorus levels (*p* < 0.001). Head circumference was modestly correlated with other anthropometric measurements, including height (Spearman correlation coefficient [*ρ*] = 0.298), weight (*ρ* = 0.359), and waist-to-hip ratio (*ρ* = 0.221) (Supplementary Table 2).

**Table 1:** Baseline characteristics of the study population

Characteristics	Overall	Sex-specific tertiles of head circumference			
		1 (smallest)	2	3 (largest)	*p*
No. of individuals	1,301	434	434	433	
Age (years), mean (SD)	57.8 (11.8)	58.9 (12.2)	57.5 (11.8)	57.1 (11.4)	0.049
Women, *n* (%)	1,032 (79.3)	344 (79.3)	344 (79.3)	344 (79.4)	1.00
Head circumference, mean (SD)	52.9 (2.1)	50.7 (1.1)	52.9 (0.8)	55.1 (1.2)	< 0.001
Height (cm), mean (SD)	158.3 (8.1)	157.5 (8.1)	158.3 (8.0)	159.3 (8.2)	0.005
Weight (kg), mean (SD)	58.4 (10.6)	55.8 (10.0)	58.2 (10.4)	61.3 (10.6)	< 0.001
BMI (kg/m^2^), mean (SD)	23.3 (3.7)	22.5 (3.5)	23.2 (3.6)	24.1 (3.7)	< 0.001
Waist-to-hip ratio	0.88 (0.13)	0.88 (0.16)	0.88 (0.11)	0.89 (0.10)	0.22
Education, *n* (%)					< 0.001
Primary or below	225 (17.3)	106 (24.4)	63 (14.5)	56 (12.9)	
Secondary	672 (51.7)	211 (48.6)	230 (53.0)	231 (53.3)	
College or university	404 (31.1)	117 (27.0)	141 (32.5)	146 (33.7)	
Ever-smoker, *n* (%)	77 (5.9)	28 (6.5)	29 (6.7)	20 (4.6)	0.37
Ever-drinker, *n* (%)	442 (34.0)	158 (36.4)	148 (34.1)	136 (31.4)	0.30
Physically inactive, *n* (%)	306 (23.5)	91 (21.0)	102 (23.5)	113 (26.1)	0.21
Family history of cancer, *n* (%)	338 (26.0)	150 (34.6)	104 (24.0)	84 (19.4)	< 0.001
Serum calcium (mmol/L)					
Mean (SD)	2.33 (0.10)	2.31 (0.09)	2.33 (0.11)	2.34 (0.09)	0.002
Missing, *n* (%)	143 (11.0)	30 (6.9)	47 (10.8)	66 (15.2)	< 0.001
Serum phosphorus (mmol/L)					
Mean (SD)	1.24 (0.16)	1.22 (0.15)	1.25 (0.17)	1.27 (0.15)	< 0.001
Missing, *n* (%)	143 (11.0)	30 (6.9)	47 (10.8)	66 (15.2)	< 0.001

*BMI* body mass index; *SD* standard deviation

**Fig. 1:**
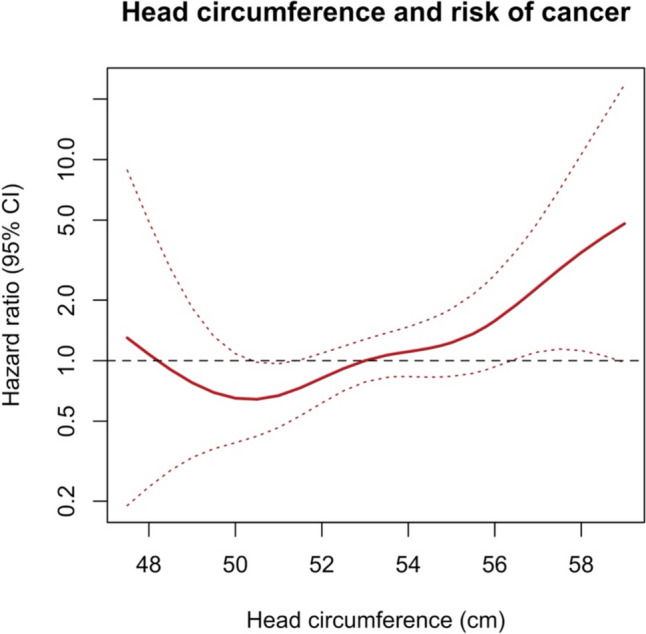
Penalized spline Cox regression for the association between head circumference and incidence of any cancer. The model was adjusted for age, sex, weight, height, education, smoking, alcohol drinking, physical activity, family history of cancer, and accounted for familial clustering. The reference used was the median head circumference of the sample (53 cm), with the hazard ratio set as 1.0

During a median follow-up of 6.9 years (IQR: 6.6–7.8 years), a total of 66 participants were diagnosed with cancer, with an incidence rate of 7.32/1,000 person-years. Table 2 shows the association between head circumference and the risk of any cancer. After adjusting for age, sex, weight, height, education smoking status, history of alcohol intake, physical activity, and family history of cancer, as well as accounting for familial clustering (model 3), every 1-cm increase in head circumference was associated with a 17% increased risk of any cancer (95% CI 1.00–1.36), which is equivalent to a 37% increased risk per SD increase (95% CI 1.00–1.87). Meanwhile, neither height nor weight was significantly associated with cancer risk after adjusting for head circumference and the other covariates in model 3 (Supplementary Table 3). The penalized spline regression curve (Fig. 1) demonstrates an increasing trend in hazard ratio with an increase in head circumference in model 3. The penalized spline regression curve also appears to suggest an increased hazard ratio for those with very low head circumference, however the hazard ratio in this region is not statistically significant with wide confidence intervals and this result should be interpreted cautiously. When analyzing head circumference as a categorical variable, individuals in tertiles 2 and 3 consistently had HRs of > 1 compared to tertile 1, although the estimates were not statistically significant, likely due to insufficient statistical power (Table 2).

**Table 2:** Association between head circumference and incidence of any cancer

Model	Per cm increase	Per SD increase	Sex-specific tertiles of head circumference		
			1 (smallest)	2	3 (largest)
No. of cases	66	66	14	26	26
Model 1^a^	1.16 (1.02, 1.33)*	1.37 (1.05, 1.79)*	1 (ref.)	1.89 (0.99, 3.64)	1.88 (0.97, 3.64)
Model 2^b^	1.17 (1.02, 1.34)*	1.37 (1.03, 1.81)*	1 (ref.)	1.85 (0.96, 3.58)	1.88 (0.96, 3.71)
Model 3^c^	1.17 (1.00, 1.36)*	1.37 (1.00, 1.87)*	1 (ref.)	1.85 (0.97, 3.52)	1.88 (0.96, 3.71)

Data are hazard ratios (95% confidence intervals) unless otherwise indicated

*p < 0.05

^a^Model 1: adjusted for age and sex

^b^Model 2: adjusted for age, sex, weight, height, education, smoking, alcohol drinking, physical activity, and family history of cancer

^c^Model 3: Model 2 + accounted for familial clustering

For the analysis of site-specific cancers, head circumference was most strongly associated with colorectal cancer (HR per cm increase: 1.81, 95% CI 1.14–2.90; per SD increase: 3.42, 95% CI 1.31–8.91) and prostate cancer (HR per cm increase: 1.58, 95% CI 1.16–2.16; per SD increase: 2.57, 95% CI 1.35–4.88), while its association with other cancers were not statistically significant (Table 3). The subgroup analyses found a significant association between head circumference and any cancer in never-drinkers (HR per cm increase: 1.38, 95% CI 1.14–1.66) (Supplementary Table 4). The association between head circumference and any cancer also appeared to be stronger in never-drinkers than in ever-drinkers (Supplementary Table 4). In the sensitivity analysis, the association between head circumference and any cancer remained similar when adjusting for waist-to-hip ratio instead of weight in model 3 or when further adjusting for serum calcium and phosphorous levels (Supplementary Table 5).

**Table 3:** Association between head circumference and risk of site-specific cancer

Outcome	No. of cases	HR per cm increase (95% CI)	HR per SD increase (95% CI)
Breast cancer (women only)	23	1.08 (0.85, 1.38)	1.17 (0.71, 1.93)
Lung cancer	11	1.25 (0.87, 1.81)	1.58 (0.74, 3.37)
Colorectal cancer	7	1.81 (1.14, 2.90)*	3.42 (1.31, 8.91)*
Stomach cancer	4	1.12 (0.63, 1.99)	1.26 (0.39, 4.10)
Prostate cancer (men only)	3	1.58 (1.16, 2.16)*	2.57 (1.35, 4.88)*

Only cancers with at least three cases were included. All the models were adjusted for age, sex, weight, height, education, smoking, alcohol drinking, physical activity, family history of cancer, and accounted for familial clustering

*p < 0.05

## Discussion

In this study, we showed a significant positive association between adult head circumference and the risk of cancer. This association did not appear to be confounded by height and weight which have also been implicated in the risk of cancer. Despite a limited sample size, we also showed a significantly increased risk of colorectal cancer and prostate cancer associated with a larger head circumference.

Previous studies [5–7] have reported associations of head circumference at birth with the development of brain cancer in childhood [5] or breast cancer [4, 6], which are in agreement with our findings. Among 1,134,143 participants recorded in the Norwegian birth registry, the relative risk of brain cancer in childhood was 1.27 (95% CI 1.16–1.38) per 1-cm increase in head circumference after adjustment for birth weight, gestational age, and sex [5]. In another study comprising 5,538 Swedish premenopausal women, those who were born with a head circumference in the top fifths were at higher risk of breast cancer in adulthood than those in the lowest fifths (HR 4.0, 95% CI 1.6–10) [4]. Adding to the literature, our study further demonstrated adult head circumference is also a significant predictor of cancer risk.

Other anthropometric measurements, such as height, have also been implicated in the risk of cancer. Height has previously been reported to be associated with cancer [15–22]. The million women study [21] found a relative risk of 1.16 (95% CI 1.14–1.17) for every 10 cm increase in height in 1,297,124 women who were followed for 11.7 million person-years. Similarly, obesity [23] and central adiposity [24] have also been associated with cancer risks. Given that head circumference is correlated with height and weight (Supplementary Table 1), it is possible that the association of head circumference with cancer is confounded by height or adiposity. Therefore, we adjusted for height, weight, as well as waist-to-hip ratio in the model, and increased head circumference remained significantly associated with an elevated risk of cancer. This is indeed in line with our recent genome-wide meta-analysis which found a nine-fold enrichment in high-fidelity cancer genes among head size genes with an intragenic lead variant, and the enrichment persisted after adjustment for height [4]. Thus, having a larger head circumference could be a marker for a higher risk of cancer. However, the underlying mechanism requires further investigation.

Similar to height, head circumference can grow continuously from infancy to childhood [25], which could be affected by hormones, especially growth hormone and insulin-like growth factors, that affect both the development of cancer and body size during childhood as well as adult life [17, 21, 26]. As all cells have the same chance of becoming cancer, an increased head circumference could contribute to the increased risk of cancer due to an increased number of cells [6]. However, in this study, we found that head circumference was associated with cancer risk independent of other measures of body size (such as height and weight), therefore, it is more likely that both head size and cancer risk are driven by an underlying shared biological mechanism. In a gene set enrichment analysis, our previous genetic study identified several enriched gene sets in cancers and signaling pathways [4]. The enriched pathways include p53, Wnt, and ErbB signaling, all of which are involved in tumorigenesis [4]. The overlapping genes in the p53 pathway are involved with the IGF-1 pathway (PTEN, IGF1), apoptosis (IGF1), and cellular senescence (CDK6, CDK2, CCND2), potentially exerting control over both cranial dimensions and cancer susceptibility. Furthermore, the overlapping genes in Wnt pathway showcases genes pivotal in bone formation (WNT3, LRP5, etc.) and oncogenic processes (APC, TP53, etc.), whereas the ErbB pathway has overlapping genes responsible for calcium signaling (PLCG1) and abnormal cell proliferation (ERBB3, AKT3) [4, 27–30]. Consequently, the genetic components within these pathways may collectively impact both cranium morphology and cancer predisposition through these mechanisms. The enriched pathways cancer types (Supplementary Table S1) include colorectal cancer and prostate cancer which were found to be significantly associated with head circumference in the current study. However, the sample size for the site-specific study was limited and cautious interpretation of the results is required. The remaining cancer types, for which no significant association was found in the current study, were endometrial cancer, basal cell carcinoma, glioma, and lung cancers (Supplementary Table S1).

Further studies are warranted to evaluate the role of head circumference in cancer pathogenesis and its management. For example, head circumference varies far more than height or weight between countries [31], it is intriguing to assess the relationship of head circumference with the prevalence of cancer in different countries. Moreover, considering the ease of measuring head circumference and its independent association with cancer risk, whereas neither height nor weight showed a significant association with cancer risk after adjusting for head circumference (Supplementary Table 3), evaluating the role of head circumference in cancer management could enhance early cancer detection and management if our findings are further confirmed in other populations.

This study has several strengths. This study is the first to explore the relationship between adult head size and cancer risk. In particular, measuring head circumference beyond infancy is uncommon, as head circumference is commonly used only as a measure of growth in children. Moreover, the cohort is linked to an electronic medical database, allowing accurate retrieval of clinical outcomes that were clinically diagnosed by physicians instead of self-reported. Furthermore, we accounted for familial clustering in the final model, although doing so did not alter the study conclusions, potentially due to the limited number of related participants. However, there are also limitations to this study. As the cohort is predominantly Chinese, female, and consists mostly of relatively healthy individuals, the results may not be generalized to other populations. Measurement errors for the head circumference are possible, although measurements were taken by trained researchers and nurses to minimize measurement bias. In addition, given the relatively small sample size and short follow-up period, only 66 events of cancer were observed in the study. The study also had insufficient power to detect most of the individual cancers, even though we demonstrated a significant association with colorectal cancer and prostate cancer. We were unable to adjust for prior cancer screening or site-specific risk factors such as inflammatory bowel disease for colorectal cancer, or reproductive factors for breast cancer due to the small sample size for each cancer. Future studies with larger samples should further study the association between head circumference and site-specific cancers in further detail. Given the small number of cancer outcomes, the analysis of the association between head circumference and site-specific cancers is exploratory and the results should be interpreted with caution. Future validation studies in different populations are warranted.

In conclusion, a larger adult head circumference was associated with an increased risk of cancer, which was independent of weight or height. Such an association could be due to shared genetics as demonstrated in a recent genome-wide meta-analysis of head circumference. Further studies are required to validate our findings in different populations and to evaluate the role of head circumference in cancer management.

## Supplementary Information

Below is the link to the electronic supplementary material.

Supplementary file1 (DOCX 316 KB)

## Data Availability

Data used in this study cannot be made publicly available due to The Personal Data (Privacy) Ordinance (Cap. 486) in Hong Kong as stated in the informed consent forms. For other requests regarding data, please contact the corresponding author (lung1212@hku.hk).

## References

[1] Bray F et al (2024) Global cancer statistics 2022: GLOBOCAN estimates of incidence and mortality worldwide for 36 cancers in 185 countries. CA Cancer J Clin 74(1):9–30 10.3322/caac.21834 38572751

[2] Emmons KM, Colditz GA (2017) Realizing the potential of cancer prevention—the role of implementation science. N Engl J Med 376(10):986–990 28273020 10.1056/NEJMsb1609101 PMC5473684

[3] Uffelmann E et al (2021) Genome-wide association studies. Nat Rev Methods Primers 1(1):59

[4] Knol MJ et al (2024) Genetic variants for head size share genes and pathways with cancer. Cell Rep Med 5(5):101529 38703765 10.1016/j.xcrm.2024.101529 PMC11148644

[5] McCormack VA et al (2003) Fetal growth and subsequent risk of breast cancer: results from long term follow up of Swedish cohort. BMJ 326(7383):248 12560272 10.1136/bmj.326.7383.248 PMC140759

[6] Samuelsen SO et al (2006) Head circumference at birth and risk of brain cancer in childhood: a population-based study. Lancet Oncol 7(1):39–42 16389182 10.1016/S1470-2045(05)70470-8

[7] Vatten LJ et al (2005) Size at birth and risk of breast cancer: prospective population-based study. Int J Cancer 114(3):461–464 15551343 10.1002/ijc.20726

[8] Cheung C-L, Tan KCB, Kung AWC (2017) Cohort profile: the Hong Kong Osteoporosis Study and the follow-up study. Int J Epidemiol 47(2):397–398f 10.1093/ije/dyx172 29024957

[9] Au PC et al (2024) The trends in lung cancer prevalence, incidence, and survival in Hong Kong over the past two decades (2002–2021): a population-based study. Lancet Reg Health West Pac 45:101030 38389934 10.1016/j.lanwpc.2024.101030 PMC10882113

[10] Lau WC et al (2017) Association between dabigatran vs warfarin and risk of osteoporotic fractures among patients with nonvalvular atrial fibrillation. JAMA 317(11):1151–1158 28324091 10.1001/jama.2017.1363

[11] Lau WCY et al (2020) Association between treatment with apixaban, dabigatran, rivaroxaban, or warfarin and risk for osteoporotic fractures among patients with atrial fibrillation: a population-based cohort study. Ann Intern Med 173(1):1–9 32423351 10.7326/M19-3671

[12] Sing CW et al (2016) Serum calcium and incident diabetes: an observational study and meta-analysis. Osteoporos Int 27(5):1747–1754 26659066 10.1007/s00198-015-3444-z

[13] Authority HKH (2016–2017) Hospital authority statistical report 2016–2017

[14] Colak A et al (2016) Correlation between calcium and phosphorus in cord blood and birth size in term infants. Minerva Pediatr 68(3):182–188 25358844

[15] Schouten LJ et al (2008) Height, body mass index, and ovarian cancer: a pooled analysis of 12 cohort studies. Cancer Epidemiol Biomark Prev 17(4):902–912 10.1158/1055-9965.EPI-07-2524 PMC2572258 18381473

[16] Olsen CM et al (2008) Anthropometric factors and risk of melanoma in women: a pooled analysis. Int J Cancer 122(5):1100–1108 17990316 10.1002/ijc.23214

[17] Gunnell D et al (2001) Height, leg length, and cancer risk: a systematic review. Epidemiol Rev 23(2):313–342 12192740 10.1093/oxfordjournals.epirev.a000809

[18] Zuccolo L et al (2008) Height and prostate cancer risk: a large nested case-control study (ProtecT) and meta-analysis. Cancer Epidemiol Biomark Prev 17(9):2325–2336 10.1158/1055-9965.EPI-08-0342 PMC2566735 18768501

[19] Pischon T et al (2006) Body size and risk of colon and rectal cancer in the European Prospective Investigation into Cancer and Nutrition (EPIC). J Natl Cancer Inst 98(13):920–931 16818856 10.1093/jnci/djj246

[20] Smith GD, Shipley M, Leon DA (1998) Height and mortality from cancer among men: prospective observational study. BMJ (Clin Res Ed) 317(7169):1351–1352 10.1136/bmj.317.7169.1351 PMC28717 9812932

[21] Green J et al (2011) Height and cancer incidence in the Million Women Study: prospective cohort, and meta-analysis of prospective studies of height and total cancer risk. Lancet Oncol 12(8):785–794 21782509 10.1016/S1470-2045(11)70154-1 PMC3148429

[22] Choi YJ et al (2019) Adult height in relation to risk of cancer in a cohort of 22,809,722 Korean adults. Br J Cancer 120(6):668–674 30778143 10.1038/s41416-018-0371-8 PMC6462046

[23] Bhaskaran K et al (2014) Body-mass index and risk of 22 specific cancers: a population-based cohort study of 5·24 million UK adults. The Lancet 384(9945):755–765 10.1016/S0140-6736(14)60892-8 PMC4151483 25129328

[24] Barberio AM et al (2019) Central body fatness is a stronger predictor of cancer risk than overall body size. Nat Commun 10(1):383 30670692 10.1038/s41467-018-08159-w PMC6342989

[25] Wright CM et al (2002) Growth reference charts for use in the United Kingdom. Arch Dis Child 86(1):11–14 11806873 10.1136/adc.86.1.11 PMC1719041

[26] Batty GD et al (2009) Height, wealth, and health: an overview with new data from three longitudinal studies. Econ Hum Biol 7(2):137–152 19628438 10.1016/j.ehb.2009.06.004

[27] Clevers H (2006) Wnt/beta-catenin signaling in development and disease. Cell 127(3):469–480 17081971 10.1016/j.cell.2006.10.018

[28] Peifer M, Polakis P (2000) Wnt signaling in oncogenesis and embryogenesis—a look outside the nucleus. Science 287(5458):1606–1609 10733430 10.1126/science.287.5458.1606

[29] Liu W et al (2014) Phospholipase Cγ1 connects the cell membrane pathway to the nuclear receptor pathway in insect steroid hormone signaling. J Biol Chem 289(19):13026–13041 24692553 10.1074/jbc.M113.547018 PMC4036317

[30] Grey W et al (2013) Deficiency of the cyclin-dependent kinase inhibitor, CDKN1B, results in overgrowth and neurodevelopmental delay. Hum Mutat 34(6):864–868 23505216 10.1002/humu.22314 PMC3708111

[31] Natale V, Rajagopalan A (2014) Worldwide variation in human growth and the World Health Organization growth standards: a systematic review. BMJ Open 4(1):e003735 24401723 10.1136/bmjopen-2013-003735 PMC3902406

