# Supplementary material for: Adult head circumference and the risk of cancer: a retrospective cohort study
Source: Cancer Causes Control. 2025 Feb 6;36(7):683–9. doi: 10.1007/s10552-025-01966-9 (PMC12103361; doi:10.1007/s10552-025-01966-9)
Supplement: Supplementary file 1 — Supplementary file1 (DOCX 316 KB) [file 10552_2025_1966_MOESM1_ESM.docx]

**Supplementary Material**


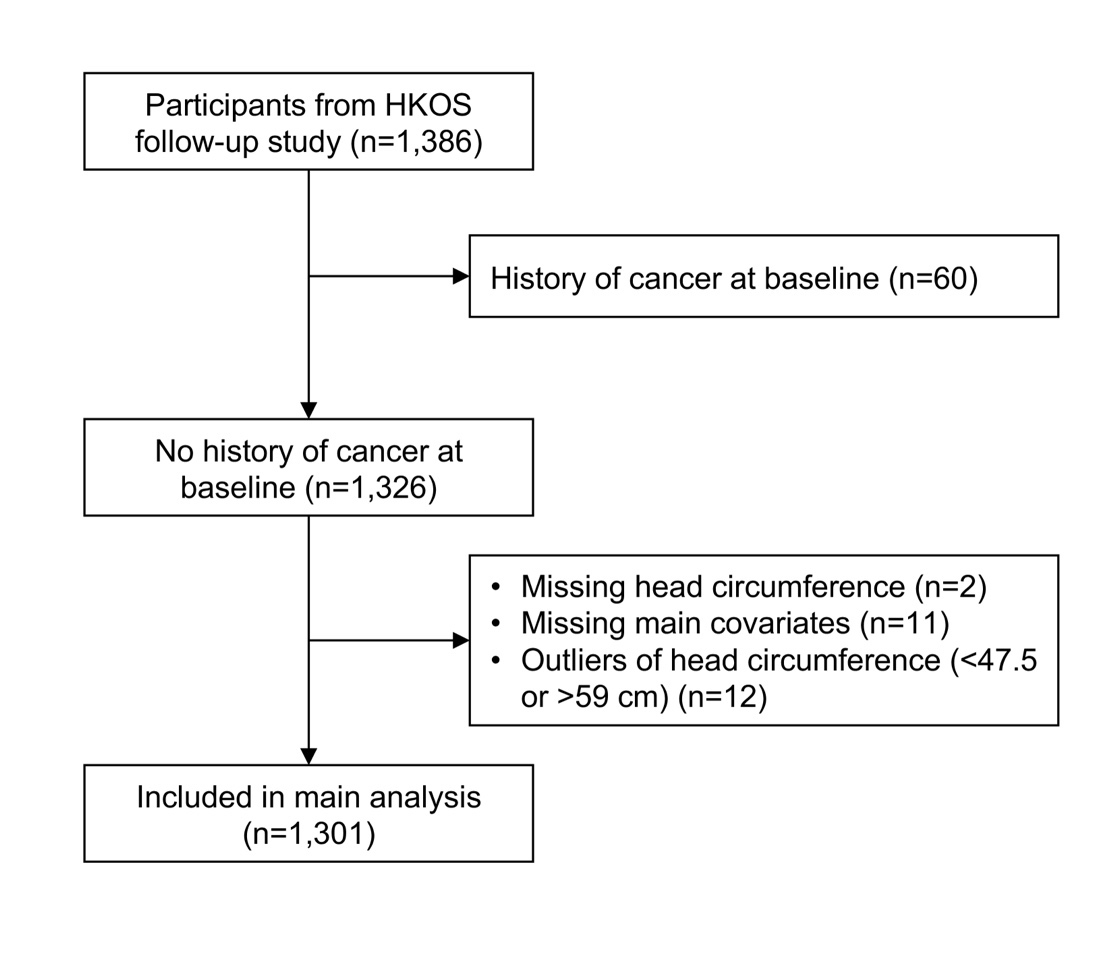


**Supplementary Figure 1.** Flowchart of sample selection.


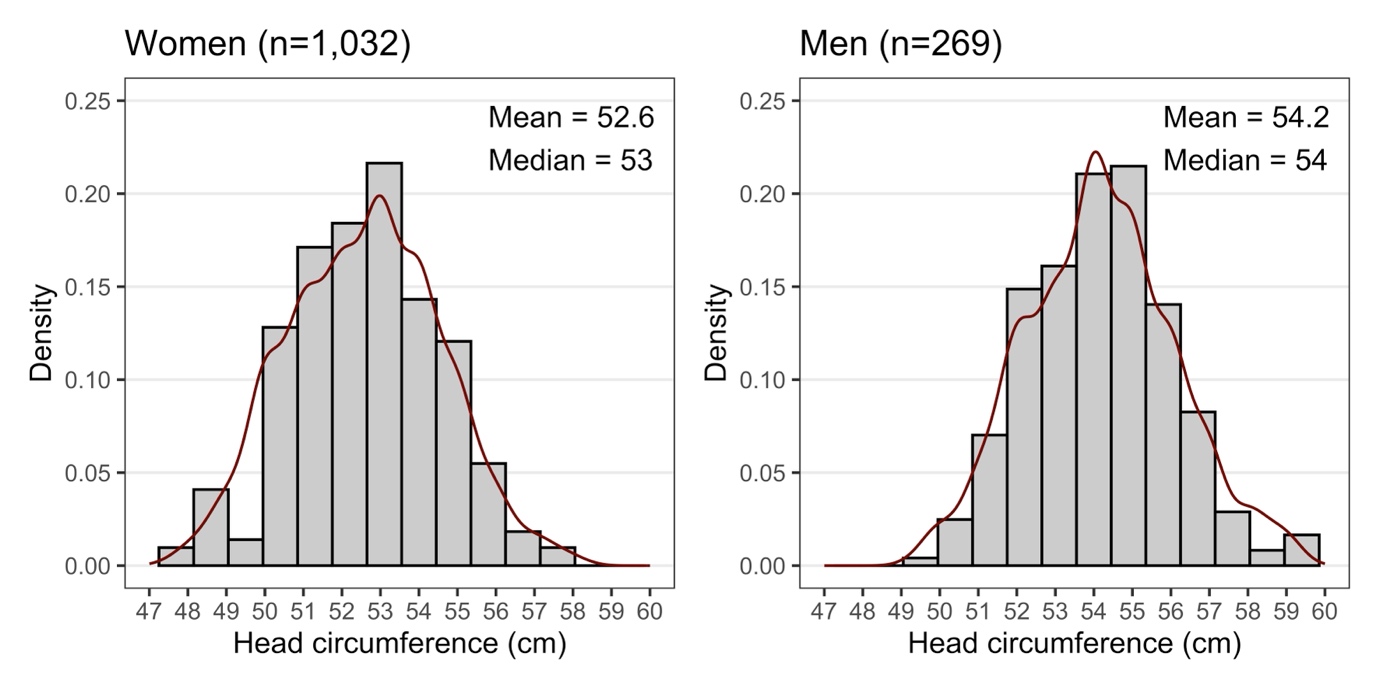


**Supplementary Figure 2.** Histogram and density plots of head circumference in the HKOS cohort, separated by sex.

**Supplementary Table 1.** Kyoto Encyclopaedia of Genes and Genomes (KEGG) pathway analysis adapted from [1].

| **GeneSet** | **No. genes** | **No. overlap** | **P-value** | **Adjusted P-value** | **Genes** |
| --- | --- | --- | --- | --- | --- |
| KEGG ENDOMETRIAL CANCER | 52 | 7 | 4.71E-06 | 0.000321 | AKT3, PTEN, TP53, TCF7L1, MAPK1, APC, FOXO3 |
| KEGG CELL CYCLE | 124 | 10 | 5.16E-06 | 0.000321 | CCND2, CDKN1B, ESPL1, CDK2, TP53, MCM2, STAG1, TFDP2, ATR, CDK6 |
| KEGG PATHWAYS IN CANCER | 325 | 16 | 5.95E-06 | 0.000321 | WNT2B, AKT3, PTEN, CDKN1B, CDK2, IGF1, TP53, FZD2, WNT3, TCF7L1, PLCG1, MAPK1, LAMB2, APC, CDK6, PTCH1 |
| KEGG BASAL CELL CARCINOMA | 55 | 7 | 6.90E-06 | 0.000321 | WNT2B, TP53, FZD2, WNT3, TCF7L1, APC, PTCH1 |
| KEGG PROSTATE CANCER | 88 | 8 | 1.92E-05 | 0.000658 | AKT3, PTEN, CDKN1B, CDK2, IGF1, TP53, TCF7L1, MAPK1 |
| KEGG GLIOMA | 65 | 7 | 2.12E-05 | 0.000658 | AKT3, PTEN, IGF1, TP53, PLCG1, MAPK1, CDK6 |
| KEGG P53 SIGNALING PATHWAY | 68 | 7 | 2.86E-05 | 0.000759 | PTEN, CCND2, CDK2, IGF1, TP53, ATR, CDK6 |
| KEGG NON-SMALL CELL LUNG CANCER | 54 | 6 | 6.99E-05 | 0.001624 | AKT3, TP53, PLCG1, MAPK1, FOXO3, CDK6 |
| KEGG SMALL CELL LUNG CANCER | 84 | 7 | 0.000112 | 0.002313 | AKT3, PTEN, CDKN1B, CDK2, TP53, LAMB2, CDK6 |
| KEGG MELANOMA | 71 | 6 | 0.000322 | 0.005995 | AKT3, PTEN, IGF1, TP53, MAPK1, CDK6 |
| KEGG WNT SIGNALING PATHWAY | 148 | 8 | 0.000722 | 0.012211 | WNT2B, LRP5, CCND2, TP53, FZD2, WNT3, TCF7L1, APC |
| KEGG ERBB SIGNALING PATHWAY | 87 | 6 | 0.000956 | 0.014823 | AKT3, CDKN1B, ERBB3, PLCG1, MAPK1, NCK1 |
| KEGG COLORECTAL CANCER | 62 | 5 | 0.001266 | 0.018109 | AKT3, TP53, TCF7L1, MAPK1, APC |
| KEGG CHRONIC MYELOID LEUKEMIA | 73 | 5 | 0.002618 | 0.034778 | AKT3, CDKN1B, TP53, MAPK1, CDK6 |

**Supplementary Table 2.** Spearman’s correlations between head circumference and age and other anthropometric measures

|  | Head circum. | Age | Height | Weight | BMI | WHR |
| --- | --- | --- | --- | --- | --- | --- |
| Head circum. | 1 |  |  |  |  |  |
| Age | -0.071 (0.010) | 1 |  |  |  |  |
| Height | 0.298 (<0.001) | -0.267 (<0.001) | 1 |  |  |  |
| Weight | 0.359 (<0.001) | -0.059 (0.035) | 0.486 (<0.001) | 1 |  |  |
| BMI | 0.213 (<0.001) | 0.112 (<0.001) | -0.072 (0.009) | 0.811 (<0.001) | 1 |  |
| WHR | 0.221 (<0.001) | 0.291 (<0.001) | 0.078 (0.005) | 0.380 (<0.001) | 0.385 (<0.001) | 1 |

BMI, body mass index; WHR, waist-to-hip ratio. Numbers in brackets are p-values.

**Supplementary Table 3.** Association of height and weight with incidence of any cancer. Data were hazard ratios (95% confidence interval) per standard deviation increase in height or weight.

|  | Height | Weight |
| --- | --- | --- |
| Fully adjusted^a^ | 1.00 (0.96, 1.05) | 1.00 (0.97, 1.03) |

1. Adjusted for age, sex, head circumference, height, weight, education, smoking, alcohol drinking, physical activity, family history of cancer, and accounted for familial clustering.

* *p*<.05

**Supplementary Table 4.** Subgroup analysis for the association between head circumference and incidence of any cancer.

| Subgroup | No. of cases | HR per cm increase (95% CI) | HR per SD increase (95% CI) | *p* for interaction |
| --- | --- | --- | --- | --- |
| Age (years) | | | | |
| <65 (n=859) | 31 | 1.10 (0.91, 1.34) | 1.22 (0.82, 1.83) | 0.11 |
| ≥65 (n=442) | 35 | 1.27 (0.99, 1.62) | 1.62 (0.98, 2.70) |  |
| Sex | | | | |
| Women (n=1,032) | 54 | 1.16 (0.99, 1.35) | 1.35 (0.98, 1.86) | 0.79 |
| Men (n=269) | 12 | 1.35 (0.82, 2.25) | 1.87 (0.66, 5.28) |  |
| Body mass index (kg/m^2^) | | | | |
| <25 (n=919) | 43 | 1.09 (0.91, 1.30) | 1.18 (0.82, 1.71) | 0.16 |
| ≥25 (n=382) | 23 | 1.32 (0.98, 1.79) | 1.78 (0.95, 3.32) |  |
| Alcohol drinking | | | | |
| Never-drinker (n=859) | 41 | 1.38 (1.14, 1.66)* | 1.93 (1.31, 2.84)* | 0.06 |
| Ever-drinker (n=442) | 25 | 0.96 (0.75, 1.23) | 0.92 (0.55, 1.52) |  |

All the models were adjusted for age, sex, weight, height, education, smoking, alcohol drinking, physical activity, family history of cancer, and accounted for familial clustering. **p*<.05

**Supplementary Table 5.** Association between head circumference and incidence of any cancer, additionally adjusted for waist-to-hip ratio, and serum calcium and phosphorus levels.

| Model | HR per cm increase | HR per SD increase | Sex-specific tertiles of head circumference | | |
| --- | --- | --- | --- | --- | --- |
|  |  |  | 1 (smallest) | 2 | 3 (largest) |
| No. of cases | 66 | 66 | 14 | 26 | 26 |
| Model 4^a^ | 1.17 (1.00, 1.36)* | 1.37 (1.01, 1.88)* | 1 (ref.) | 1.88 (0.98, 3.58) | 1.92 (0.98, 3.78) |
| Model 5^b^ | 1.15 (0.99, 1.36) | 1.35 (0.98, 1.88) | 1 (ref.) | 1.97 (1.01, 3.84)* | 1.94 (0.94, 3.99) |

1. Model 4: adjusted for age, sex, waist-to-hip ratio, height, education, smoking, alcohol drinking, physical activity, family history of cancer, and accounted for familial clustering.
2. Model 5: adjusted for age, sex, weight, height, education, smoking, alcohol drinking, physical activity, serum calcium and phosphorus levels, family history of cancer, and accounted for familial clustering. Sample size for model 5 was smaller due to missing data for calcium and phosphorus (n=1158, incident cases=59).

* *p*<.05

1. Knol, M.J., et al., *Genetic variants for head size share genes and pathways with cancer.* Cell Rep Med, 2024. **5**(5): p. 101529.
